# Bilateral, Unaugmented, Loop Myopexy Performed for a Severe Case of Heavy Eye Syndrome

**DOI:** 10.22599/bioj.125

**Published:** 2019-02-15

**Authors:** David Maskill, Janice Hoole, Katerina Oikonomi, Ian Simmons, Evangelos Drimtzias

**Affiliations:** 1 St James University Hospital, LEEDS Teaching Hospitals, GB; 2 Patras Olympion General Clinic, GR

**Keywords:** Heavy eye syndrome, Progressive esotropia

## Abstract

**Aim:**

To report the clinical features and surgical outcomes of one patient with heavy eye syndrome who underwent bilateral, unaugmented, full loop myopexy.

**Methods:**

A 47-year-old lady with high myopia, high axial length, progressive esotropia, slippage of the lateral rectus (LR) inferiorly and superior rectus (SR) medially on magnetic resonance imaging (MRI) was diagnosed with heavy eye syndrome. Unaugmented loop myopexy without medial rectus (MR) recession was offered.

**Results:**

On follow-up at 30 months, a small residual esotropia of 6 prism diopters (PD) at near and 10 PD at distance was achieved. Both abduction and elevation were improved in both eyes.

**Conclusions:**

The high angle of esodeviation can be challenging to correct adequately with surgery, with many options available: resection-recession, hemitranspositions (Yamada’s procedure), partial loop myopexy (modified Jensen’s procedure) and full loop myopexy (Yokoyama’s procedure). It remains unclear which procedure is optimal for severe disease. In this case, we present bilateral, unaugmented, full loop myopexy as our preferred choice for high esotropia.

## Introduction

The supertemporal quadrant between the SR and LR muscles lacks any extraocular muscles. The intermuscular membrane is the only supporting tissue giving way to the expanding force of the globe when is too large to fit within the muscle cone because of axial high myopia (Yamaguchi, Yukoyama & Shiraki 2010). This results in superotemporal dislocation of the posterior portion of the elongated globe out from the muscle cone (Yamaguchi, Yukoyama & Shiraki 2010). As a consequence, eye movements become restricted in abduction and supraduction with subsequent progressive esotropia. This extreme condition has been called heavy eye syndrome. Previous authors hypothesized that the esotropia described is due to the weakening of the lateral rectus muscle from prolonged near work (Zheng et al. 2018). We present one case with heavy eye syndrome who underwent bilateral, unaugmented, full loop myopexy.

## Case report

A 47-year-old lady was seen in the ocular motility clinic with a longstanding history of progressively increasing large-angle esotropia since early childhood, otherwise asymptomatic.

On examination, visual acuity was 0.8 LogMAR in the right eye and 0.8 LogMAR in the left. Ocular refraction was –24.00 D in the right eye and –25.00 D in the left eye. Anterior segment examination showed tiny dot cataracts in either eye of no clinical significance. Her fundus examination showed changes compatible with pathologic axial myopia.

**Figure 1:**
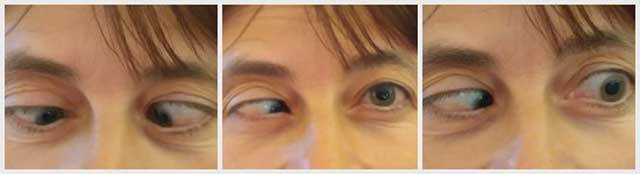
Primary position, right and left gaze of our patient at presentation.

She had a marked right esotropia with hypotropia measuring 70 prism diopters base out and 10 prism diopters base up in the right eye with the help of Krimsky’s test. Both abduction and elevation were limited to –7.00 and –5.00 respectively in the right eye and to –4.00 and –3.00 in the left. In contrast, both adduction and infraduction were found to be intact in either eye. Figure 1 shows primary position, right and left versions in our patient.

**Figure 2:**
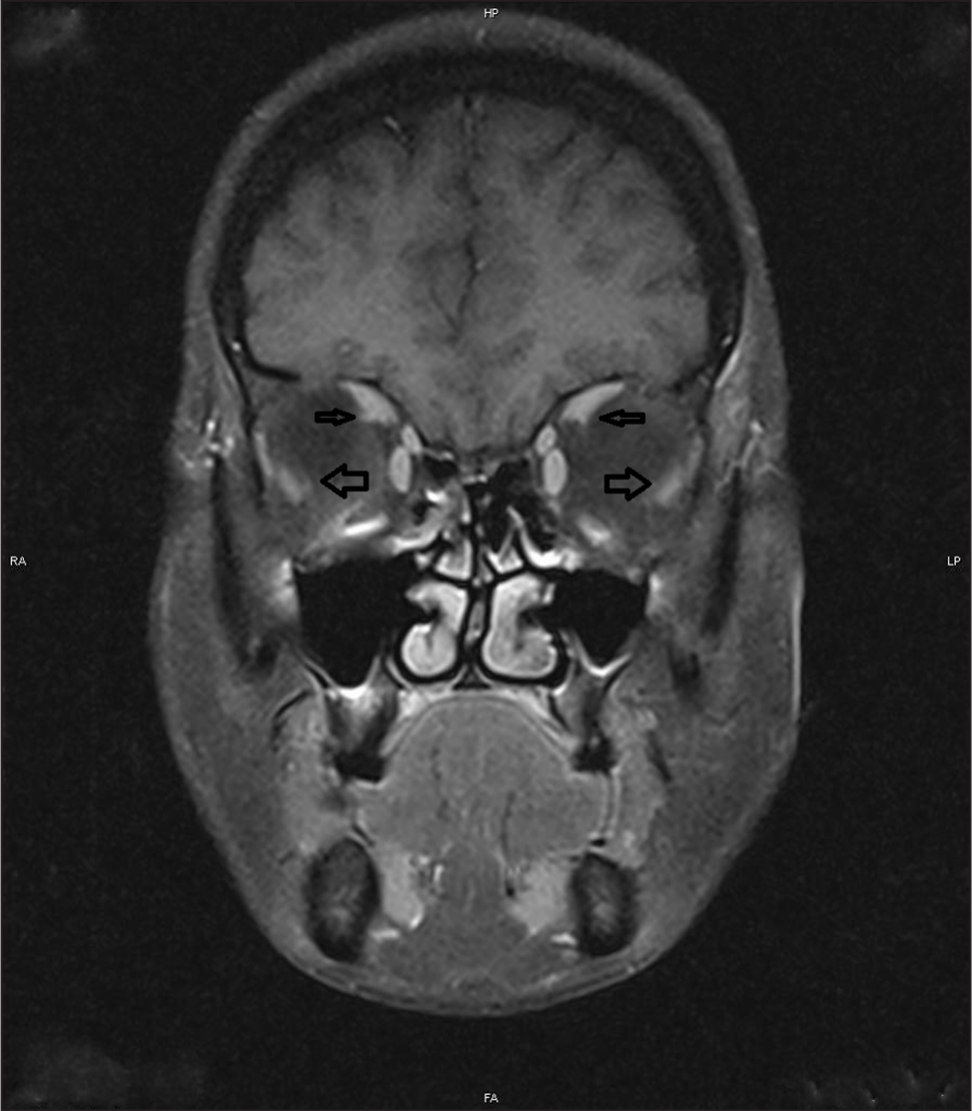
Magnetic resonance imaging of the orbit showing superotemporal herniation of the eyeball with inferior displaced lateral rectus (thick arrows) and nasally displaced superior rectus muscles (thin arrows).

Subsequent MRI excluded any neuropathology, instead demonstrating inferior displacement of the LR and nasal displacement of the SR in both eyes (Figure 2). The patient was therefore diagnosed with myopic strabismus fixus, also known as heavy eye syndrome.

Surgery was done under general anesthesia. A forced duction test (FDT) was performed and was found to be negative showing no evidence of MR tightness in either eye. Therefore, recession of MR was considered to be inappropriate. A loop myopexy between SR and LR was performed to correct the path of the LR and SR. A superior and temporal limbal peritomy was performed and both the superior and lateral recti muscles were identified, dissected and isolated with a 4–0 silk suture (Mersilk, Ethicon). The temporal half of the SR muscle and the superior half of the LR muscle were sutured with a non-absorbable suture (ethibond 5–0) and were then united together at two sites, 5 mm and 7 mm from their insertions without any attachment to the sclera. The conjunctiva was sutured with 8–0 vicryl. The operation was performed bilaterally.

**Figure 3:**
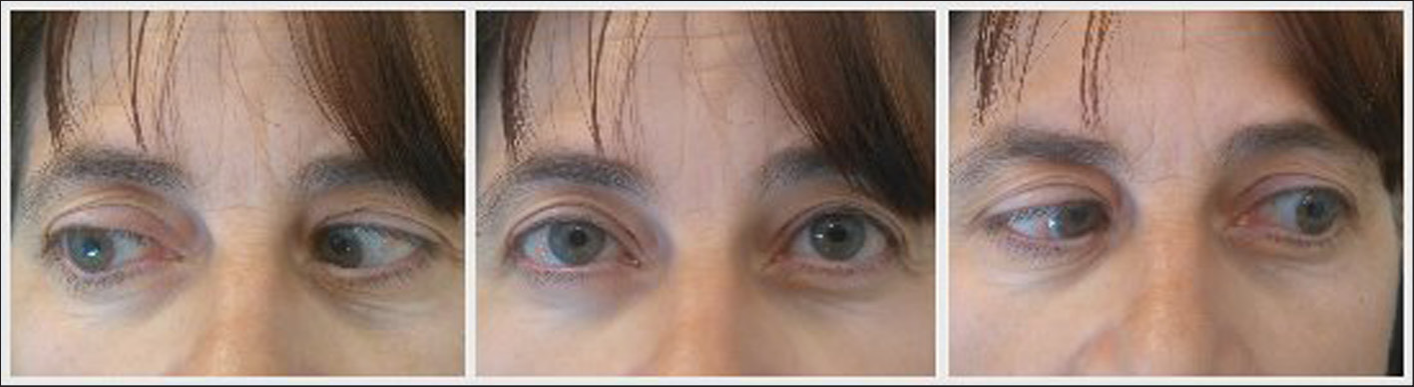
Primary position, right and left gaze at last review.

The patient was nearly orthophoric the day after the operation. On follow-up at 30 months, a small esotropia of 6 PD at near and 10 PD at distance along with a small right hypotropia of 3 PD for both near and distance was achieved. Both abduction and elevation were improved in both eyes though were remained slightly limited (–2 respectively in either eye) as shown in Figure 3. Overall, the patient was satisfied with the result.

## Discussion

For small-angle deviations of myopic strabismus fixus, combined recession-resection procedures can be sufficient (Sturm et al. 2008). With more severe abduction deficits however, as in the presented case, this approach results in poor long-term outcomes. Once MRI imaging has identified the altered courses of the extraocular muscles, it becomes easier to aim for normalisation of the force vectors. The loop myopexy--unification of the SR and LR--is an effective procedure for this purpose (Yamaguchi, Yokoyama & Skiraki 2010). Efforts to optimise this procedure have led to a range of surgical techniques. The simplest modification investigated was that of scleral fixation, but this was shown to be ineffective (Lam et al. 2015). The implantation of gore-tex slings or silicon band loops in place of sutures has also been trialled but with limited success due to foreign body sensations (Shenoy, Sachdeva & Kekunnaya 2015; Shih, Li & Huang 2012), further hampered by the challenging nature of these lengthier and costlier procedures. One study suggested the insertion of three sutures rather than one, hypothetically strengthening the union of the SR and LR, conferring extra strength and less restriction of movement (Farid, Ebarky & Saeed 2016). This study showed the superiority of the three-suture technique but attained worse results for the single-suture technique than have been reported in previous studies (Akar et al. 2014; Zou et al. 2017), likely due to a large difference in preoperative angles of deviation. This casts doubt on their comparison. Previous study suggested that additional MR recession may be effective in some patients but relapse can occur (Sturm et al. 2008). No MR recession was necessary in the present case. Indeed, a study of unaugmented loop myopexy without MR recession has demonstrated that muscle union alone is an effective treatment (Bansal & Marsh 2016). The present case serves to strengthen this argument.

## Conclusion

Muscle union surgery is an effective procedure for heavy eye syndrome. This surgery can normalize the vectors of muscle force of the SR and LR restoring the dislocated globe back into the muscle cone. MR recession is not essential, however this would be obligatory when contracture of the MR is suspected during surgery.
